# The association between heightened ADHD symptoms and cytokine and fatty acid concentrations during pregnancy

**DOI:** 10.3389/fpsyt.2022.855265

**Published:** 2022-07-22

**Authors:** Hanna C. Gustafsson, Geoffrey A. Dunn, A. J. Mitchell, Kathleen F. Holton, Jennifer M. Loftis, Joel T. Nigg, Elinor L. Sullivan

**Affiliations:** ^1^Department of Psychiatry, Oregon Health & Science University, Portland, OR, United States; ^2^Department of Human Physiology, University of Oregon, Eugene, OR, United States; ^3^Department of Behavioral Neuroscience, Oregon Health & Science University, Portland, OR, United States; ^4^Departments of Health Studies and Neuroscience, American University, Washington, DC, United States; ^5^VA Portland Health Care System, Portland, OR, United States; ^6^Division of Neuroscience, Oregon National Primate Research Center, Beaverton, OR, United States

**Keywords:** ADHD, pregnancy, pro-inflammatory cytokines, tumor necrosis factor-α, interleukin-6 (IL-6), omega-3 fatty acids (n-3), omega-6 fatty acids (n-6)

## Abstract

**Objective:**

Previous research conducted with samples of children suggest that individuals with attention-deficit/hyperactivity disorder (ADHD) have altered fatty acid concentrations and may have increased systemic inflammation. Whether these differences are also apparent in other populations of individuals with heightened ADHD symptoms (e.g., pregnant adults) is unknown. The goal of the current study was to examine whether there are ADHD-associated differences in polyunsaturated fatty acid concentrations or pro-inflammatory cytokine concentrations during pregnancy, a developmental period when fatty acid concentrations and systemic inflammation have implications for the health of both the pregnant person and the developing child. We hypothesized that plasma levels of the ratio of omega-6s to omega-3s (n-6:n-3) and plasma inflammatory cytokine levels would be higher in individuals with heightened ADHD symptoms, consistent with previous findings in children with ADHD.

**Methods:**

Data (*N* = 68) came from a prospective study of pregnant community volunteers who were oversampled for ADHD symptoms. During the 3rd trimester, plasma concentrations of fatty acids and the pro-inflammatory cytokines interleukin-6 (IL-6) and tumor necrosis factor-alpha (TNF-α) were assessed. Dietary intake was examined in the 3rd trimester using three 24-h recalls conducted by trained dietitians and by examining plasma levels of conjugated linoleic acid (n-6) and α-linolenic acid (n-3), essential fatty acids that must come from dietary intake.

**Results:**

The group with heightened ADHD symptoms had higher n-6:n-3s (β = 0.30, *p* < 0.01) and higher TNF-α concentrations (β = 0.35, *p* < 0.001) relative to controls. There were no group differences in dietary variables, as assessed by self-report and *via* plasma concentrations of essential fatty acids. IL-6 was not reliably associated with ADHD status in this sample.

**Conclusion:**

Pregnant individuals with ADHD, on average, had higher plasma n-6:n-3s and higher TNF-α concentrations relative to controls. A difference was not detected in their dietary intake of fatty acids or other relevant nutrients. Though these null findings are inconclusive, they are consistent with the hypothesis that ADHD-associated differences in plasma fatty acid concentrations are the result of ADHD-associated differences in fatty acid metabolism, rather than simply differences in dietary intake.

## Introduction

Interest in the nutritional correlates of attention-deficit/hyperactivity disorder (ADHD) has grown over the last two decades, with substantial attention paid to the role of polyunsaturated fatty acids (PUFAs). Most of this research has focused on whether omega(n)-3 fatty acid levels, either consumed in the diet or measured in circulation, differ between individuals with and without ADHD. Results generally suggest that ADHD is associated with lower plasma n-3 fatty acid concentrations (1–3) however differences in fatty acid concentrations between ADHD and control groups often are small in magnitude (1) and n-3 supplement trials aimed at treating ADHD have reported mixed results (1, 3–5). One proposed reason for the small observed differences between ADHD and control groups (1, 6) is that consideration of circulating levels of n-3 fatty acids alone is not a sufficiently comprehensive indicator of fatty acid levels, as it does not take into account concentrations of n-6 fatty acids, which compete for the same enzymes for elongation and desaturation (7). Thus, absolute levels of n-3 or n-6 fatty acid concentrations may not be as meaningful of a metric as the ratio of n-6 to n-3 fatty acids (n-6:n-3). A greater n-6:n-3 has been linked with increased systemic inflammation and with increased risk for diseases with inflammatory underpinnings (8–10), though few studies have empirically examined blood levels of fatty acids and inflammatory markers together in the context of psychiatric disorders [see (11, 12) for exceptions], and none to our knowledge have investigated these associations among individuals with heightened symptoms of ADHD.

Research looking at the n-6:n-3 in individuals with and without ADHD is more limited than studies of n-3s alone (there have been fewer than 10 studies to date), but these studies also support a picture of altered fatty acid status in ADHD (6, 13, 14). As is true for the n-3 studies, there is some heterogeneity in the sample types used (e.g., plasma vs. serum vs. red blood cells) as well as in which fatty acids were used to compute the n-6:n-3. Most studies report the ratio of all measured n-6s to n-3s (the specific fatty acids considered varies from study to study), while some also report the ratio of arachidonic acid (AA; an n-6) to eicosapentaenoic acid (EPA; an n-3; AA:EPA), selected because they are precursors to important inflammatory mediators (15) and because they are important biologically active n-6 and n-3 fatty acids in the brain (6). With these study design differences aside, the extant studies typically report that the n-6:n-3 is increased in individuals with ADHD, even in the absence of differences in absolute values of n-3s or n-6s [for exceptions see (4, 16)]. For example, Parletta et al. (8), using a sample of children from Melbourne, Australia, found that the AA:EPA and the n-6:n-3 in red blood cells were elevated in children with ADHD relative to controls (mean age = 9.10 years; 80% male). Utilizing data from a sample of children living in Taiwan, Wang et al. (13) found that the n-6:n-3 was elevated in the serum of children with ADHD relative to controls (mean age = 9.20 years; 86% male). In another study, Stevens et al. (17), using a US sample (mean age = 9.10 years; 100% male), found that males with ADHD had greater plasma (but not red blood cell) n-6:n-3s, even though they did not differ in their absolute levels of n-6 fatty acids. Though these studies offer important preliminary information about the n-6:n-3 and ADHD, a number of important questions remain.

First, while the previous studies on this topic are informative, they have been conducted almost exclusively with samples of children or adolescents and have utilized data from samples that are predominantly male. While this focus on male youth is consistent with the known demographics of ADHD [which is more common in males (18) and is typically diagnosed in childhood], these demographics are not representative of all individuals with ADHD, and thus it is unclear if these results generalize to other populations with ADHD (e.g., adult females). ADHD is a neurodevelopmental disorder that is commonly diagnosed in childhood, however in about two-thirds of cases, clinically significant or impairing symptoms persist into adulthood (19, 20), making this an important population to study. Evaluating whether other subgroups of individuals with ADHD also show evidence of altered fatty acid concentrations is important, as this information may give further insight into the etiology and pathophysiology of ADHD, and because this information may help with the design of treatments that have potential to help a wider range of individuals.

Arguably, if there were to be a group of individuals with ADHD that these results would be least likely to extend to, it would be pregnant individuals. Previous research shows that there are sex differences in fatty acid synthesis that are the result of sex hormone-induced alterations in the expression of desaturase and elongase enzymes (21, 22); these differences, however, have typically been studied in non-pregnant populations. They are likely to be exaggerated during pregnancy given the normative and marked increase in sex-hormones that accompanies the pregnant state (23). Indeed, pregnancy is typically characterized by hyperlipidemia (24, 25), which appears to intensify in correspondence with pregnancy-related hormonal changes, and may be responsible for the depletion in long-chain fatty acid concentrations that appears to occur starting in the second trimester (26–28). These pregnancy-specific changes in fatty acid metabolism make our primary research question (i.e., whether the n-6:n-3 differs in pregnant individuals with and without ADHD) a non-trivial extension of previous studies in this area.

Second, there exists ambiguity as to whether ADHD-associated differences in the n-6:n-3 in plasma reflect differences in fatty acid metabolism or if they are due to ADHD-associated differences in dietary intake (either in the form of diet or n-3 supplementation). One hypothesis is that these disparities are the result of metabolic differences (29); this hypothesis is supported by research that suggests that there are ADHD-associated differences in fatty acid desaturase genes and phospholipid metabolism genes (30, 31). However, few studies have examined the n-6:n-3 in ADHD alongside measures of dietary intake, and the existing studies have yielded somewhat mixed results. While two studies (4, 32) suggest that youth with and without ADHD do not differ in their dietary intake of relevant fatty acids, at least one study reported that children with ADHD consumed less healthful foods, including more frequent consumption of high-fat and high-sugar foods, assessed using a food frequency questionnaire (13). This question has not yet been addressed during pregnancy, a time when dietary patterns may change to accommodate the nutritional requirements needed to support the developing child, and that may change in response to physiologic changes associated with food aversions, cravings, nausea, vomiting, tiredness, and heartburn (33). Importantly, the circulating fatty acid concentrations of the pregnant individual has implications for the health of both the pregnant person and the developing fetus (26–28).

To examine dietary-intake differences in individuals with and without ADHD, the gold standard is 24-h recall data conducted by trained dietitians. A second source of information about dietary intake comes from examining blood levels of essential fatty acids (those that cannot be synthesized by the human body, and thus must come from diet). Most relevant here are the essential fatty acids linoleic acid (n-6) and α-linolenic acid (n-3) (34). If n-6:n-3 differences in plasma are observed in the absence of differences in dietary intake (assessed either *via* recalled dietary intake or by concentrations of essential fatty acids), this would be consistent with the hypothesis that there are ADHD-associated differences in the metabolism rather than the consumption of fatty acids.

The third remaining question revolves around the relations among fatty acid concentrations, systemic inflammation, and ADHD. Increased systemic inflammation has been implicated in the etiology and pathophysiology of ADHD (35–37), yet limited empirical attention has been paid to the association between ADHD and inflammation in humans. The evidence that does exist generally suggests that, relative to controls, children (38–40) with ADHD have higher circulating levels of pro-inflammatory cytokines, including interleukin (IL)-6 and tumor necrosis factor-alpha (TNF-α). Whether these differences in cytokines are also apparent in pregnant individuals with ADHD remains untested, despite evidence that these cytokines are elevated in the context of other psychiatric symptoms during pregnancy (e.g., depression) (41, 42). There are normative changes in immune system functioning that occur during pregnancy to support healthy fetal growth and development (43, 44)), which again makes it challenging to extend the findings of previous research to this population. Additionally, although the n-6:n-3 has been shown to be related to greater inflammation in non-pregnant adults (45–47), its association with inflammation in pregnancy, and in the context of ADHD, has not yet been explored. This is important because it would have implications for both intervention and even for theories of mechanisms of intergenerational transmission.

To address these outstanding questions, the goal of the current study was to test:

1. Does the ratio of n-6s to n-3s in plasma differ in pregnant individuals with and without heightened symptoms of ADHD?
2. Are there differences in dietary intake of fatty acids (assessed using 24-h dietary recall and/or essential fatty acid concentrations) between individuals with and without heightened symptoms of ADHD?
3. Do pregnant individuals with heightened symptoms of ADHD have higher plasma cytokine concentrations (IL-6 and TNF-α) than controls? Is the ratio of n-6s to n-3s in plasma related to greater cytokine concentrations?

We hypothesized that, on average, individuals with heightened symptoms of ADHD would have higher plasma n-6:n3s and higher plasma concentrations of IL-6 and TNF-α. We also hypothesized that a higher n-6:n-3 would be positively correlated with inflammation. Consistent with the hypothesis that there are fatty acid conversion or metabolic differences in ADHD, we hypothesized that there would be no ADHD-associated differences in dietary intake or in essential fatty acid concentrations.

In testing our hypotheses, we controlled for whether the pregnant individual reported that they were currently consuming a fish oil or fatty acid supplement. In models that considered inflammation, we also controlled for pre-pregnancy adiposity. These variables were selected as covariates *a priori*, given presumed effects of fatty acid supplementation on plasma fatty acid concentrations, and given the large literature linking adiposity and pro-inflammatory cytokine concentrations (48, 49).

## Methods

### Participants and procedures

Data came from a prospective longitudinal study described previously (50, 51). The overarching goal of this study was to investigate prenatal and early life influences on offspring risk for psychopathology. Pregnant individuals (*n* = 62) were recruited through an urban hospital-based outpatient prenatal clinic in the second trimester of pregnancy and were followed into the postpartum period. After enrolling in the study with the first target child, six participants conceived a second child and completed all of the same assessments for their second pregnancy, resulting in data on six sibling pairs and yielding a final analytic sample of *N* = 68). This nesting of children within families was handled statistically (see Analytic Strategy for details).

To serve the goals of the overarching project, an effort was made to oversample for individuals who endorsed a current or childhood history of ADHD or high levels of current ADHD symptoms (see full description of ADHD status definition below). Exclusion criteria included high-risk or medically complicated pregnancy, extreme life circumstances (specifically, homelessness), being <18 years old, and active substance use (including alcohol, tobacco, marijuana, opioids, and cocaine). Data used in the current analyses came from laboratory visits when participants were 37 weeks pregnant. At this visit, participants provided a blood sample and reported on demographic information *via* surveys. Participants also reported on their dietary intake within 2 weeks of their laboratory visit. Participant medical records were reviewed for information about their health. Oregon Health & Science University's (OHSU) Institutional Review Board approved all procedures, and written informed consent was obtained from all participants.

### Measures

#### Demographics

Pregnant person age (years) and highest completed education (1 = Grade School, 2 = Some High School, 3 = High School Equivalent, 4 = High School Degree, 5 = Some College but No Degree, 6 = Associates Degree, 7 = Bachelor's Degree, 8 = Masters, Law, 2–3 years degree, 9 = Doctorate, PhD, Medical Degree) were self-reported.

#### ADHD status

Pregnant person ADHD status was defined as a current or childhood history of ADHD or current elevated symptoms of ADHD, defined as >80th percentile on the Barkley Adult ADHD Rating scale (BAARS-IV) Quick Screen (52). Fifty-three percent of participants (*n* = 36) met this criterion. In this manuscript, we will refer to these individuals as the heightened ADHD symptoms (vs. control) group, given their history of or current levels of significant ADHD symptomatology, though we recognize that this shorthand does not adequately capture all of the nuance of our measurement.

#### Plasma fatty acids

Plasma fatty acids were assessed using blood samples collected in the 3rd trimester (34–37 weeks gestation). Blood was drawn by venipuncture into K_2_ EDTA tubes (BD Vacutainer Systems, Franklin Lakes, NJ), centrifuged, and plasma was separated, aliquoted, and frozen at −80°C until assay. Plasma fatty acids were analyzed by direct transesterification using a Trace GC coupled to a DSQ mass spectrometer (ThermoElectron) as described previously (53). N-6 fatty acids included in this study were conjugated linoleic acid, γ-linolenic acid (C18:3), dihomo-γ-linolenic acid (C20:3), and arachidonic acid (AA; C20:4). N-3 fatty acids included in this study were α-linolenic acid (C18:3), eicosapentaenoic acid (EPA; C20:5), and docosahexaenoic acid (C22:6). The n-6:n-3 was captured two ways: (1) by summing all of the n-6 fatty acid concentrations and dividing that total by the sum of all of the n-3 fatty acid concentrations (n-6:n-3), and (2) the AA:EPA, calculated by dividing arachidonic acid concentrations by eicosapentaenoic acid concentrations.

#### Pre-pregnancy adiposity

Participants' medical records were reviewed for pre-pregnancy body mass index (BMI; kg/m^2^).

#### Inflammation

Pregnant person inflammation was assessed using 3rd trimester plasma concentrations of IL-6 and TNF-α. Blood used for these assays was collected at the same time as the blood used in the fatty acid assay (described above); blood was drawn, processed, and stored in the same manner. Blood draws were scheduled around participant prenatal care appointments. While this helped to minimize participant burden, it precluded our ability to completely standardized the exact time of day that the blood draw occurred. While most study visits were collected in the early to mid-morning, not all were.

Plasma concentrations of IL-6 were measured by enzyme-linked immunosorbent assays (Human IL-6 quantikine HS ELISA kits (HS600B; assay range: 0.2–10 pg/ml, sensitivity: 0.11 pg/ml), R&D Systems, Inc., Minneapolis, MN) according to the manufacturer's instructions and as described previously (41). All standards and samples were run in duplicate. Briefly, plasma samples were initially diluted 1:2 using Assay Diluent RD1-75 and incubated for 2 h at room temperature on a horizontal orbital microplate shaker. Following standard wash procedures, human IL-6 HS Conjugate was added to each well, and plates were incubated as described above. Plates were then washed and incubated with the Substrate Solution (60 min), Amplifier Solution (30 min), and Stop Solution. Plates were read within 30 min of adding the Stop Solution using a microplate spectrophotometer (Benchmark Plus microplate, Bio-Rad Laboratories, Inc., Hercules, CA).

Plasma concentrations of TNF-α (assay range: 5.3–3,900 pg/ml, sensitivity: 1.5 pg/ml) were assayed using Luminex polystyrene bead-based multiplex immunoassays (customized Luminex Performance Human Obesity Panel, FCST08-05; R&D Systems) according to the manufacturer's instructions and as described previously (41). This panel also assessed concentrations of monocyte chemoattractant protein-1 (MCP-1), however these values were not considered in this study because this cytokine has not been implicated in previous research comparing children with and without ADHD. All standards and samples were run in duplicate. Plasma samples were initially diluted 1:2 using the matrix solution provided, and samples were incubated overnight at 4°C with color-coded beads that were pre-coated with cytokine-specific capture antibodies. The plates were then washed by vacuum filtration, incubated with biotinylated detection antibodies (1 h, room temperature), washed, and incubated with phycoerythrin-conjugated streptavidin (30 min, room temperature). Plates were read on the dual-laser, flow-based Luminex 100 Analyzer (Luminex, Austin, TX). For both the ELISAs and multiplex assays, sample values were determined based on standard curves calculated using computer software to generate four- and five-parameter curve-fits, respectively (Prism 7 for Windows, GraphPad Software, Inc., La Jolla, CA).

#### Dietary intake

Diet during pregnancy was measured by three, non-consecutive 24-h diet recalls that were conducted over the phone by trained dietitians in the 3rd trimester of pregnancy. Trained staff, overseen by a dietitian certified in the procedure, used the multi-pass method (54) and Nutrition Data System for Research Software, developed by Nutrition Coordinating Center (University of Minnesota, Minneapolis, Minnesota) to record the previous day's food and drink intake and supplement use over the previous 30 days. The software facilitates the collection of recalls in a standardized fashion in a multiple-pass interview approach. The recalls were unscheduled and unannounced, including 1 weekend day and 2 weekdays, and took place over a 2-week period. At each time point, the dietitian inquired about food intake the day prior. All interviewers completed a training program and met qualification standards established in the Oregon Clinical and Translational Research Institute Bionutrition Unit using the Nutrition Data System for Research (NDSR) software (Nutrition Coordinating Center (NCC), University of Minnesota). Nutrient values were averaged across the three recalls to create average dietary intake values. In the current study, we examined total calories (kcal), total fat (g), percent of calories from fat (%), total polyunsaturated fatty acids (g), total n-3 fatty acids (g), saturated fatty acids (SFA) (g), monounsaturated fatty acids (MUFA) (g), and the PUFA-to-SFA ratio (PUFA:SFA).

Whether the participant reported taking a fish oil or fatty acid supplement during the previous 30 days (0 = not taking a supplement, 1 = taking a supplement) was also recorded. Individuals who endorsed taking a fatty acid supplement typically reported taking a supplement that contained n-3 fatty acids (e.g., DHA, EPA), though the specific ingredients varied between participants. Forty-one percent of participants reported taking a fish oil or fatty acid supplement in the previous 30 days.

### Analytic strategy

Research questions were tested using M*plus* 8.5 (Muthén and Muthén, 1998–2017) using the robust maximum likelihood estimator. This estimator can accommodate non-normally distributed data. Missing data were handled using full information maximum likelihood (55). Non-independence of observations (i.e., the nesting of children within families) was handled using the M*plus Cluster* command.

Descriptive statistics (means, standard deviations, and percentages) for all study variables are presented in Tables 1, 2. In Table 2, we present raw cytokine concentrations for ease of interpretation, however these values were log-transformed prior to all analyses. In preliminary analyses, we compared the ADHD and control groups on all study variables using independent samples *t*-tests. These preliminary analyses were followed by formal hypothesis testing, which involved examining these same group differences while controlling for relevant confounds and accounting for the nesting of children within families.

**Table 1:** Sample demographic information (*N* = 68).

	**Mean (SD)**	**Mean (SD)**	**Mean (SD)**
	**Total (*****N*** = **68)**	**Control (*****n*** = **32)**	**ADHD (*****n*** = **36)**
Pregnant person age (Years)	30.49 (5.00)	31.77 (5.07)	29.35 (4.82)^+^
Pre-pregnancy body mass index (BMI)	26.85 (6.83)	23.58 (2.73)	29.12 (8.04)**
Highest completed education^a^	6.72 (1.20)	7.41 (0.97)	6.15 (1.06)**
Average BAARS-IV score	9.98 (4.21)	6.41 (1.45)	13.03 (3.29)**

*The ADHD and Control groups were compared to one another using t-tests. Significant and marginally significant groups differences based on these analyses are denoted in the table:*

+ *p < 0.10,*

** *p < 0.01.*

a *Education was reported as follows: 1 = Grade School, 2 = Some High School, 3 = High School Equivalent, 4 = High School degree, 5 = Some College but no degree, 6 = Associates degree, 7 = Bachelor's degree, 8 = Masters, Law, 2–3 years degree, 9 = Doctorate, PhD, Medical degree. BAARS-IV, Barkley Adult ADHD Rating scale Quick Screen*.

**Table 2:** Means for all study variables (*N* = 68).

	**Mean (SD) or %**	**Mean (SD) or %**	**Mean (SD) or %**
	**Total (*****N*** = **68)**	**Control (*****n*** = **32)**	**ADHD (*****n*** = **36)**
Pre-pregnancy body mass index (kg/m^2^)	26.77 (6.88)	23.58 (2.72)	29.12 (8.04)
**3rd Trimester Plasma Fatty Acid Levels**			
*n-3*			
Sum of omega-3 fatty acids (nmol/ml)	532.51 (171.79)	538.45 (179.69)	526.87 (168.42)
α-linolenic acid (nmol/ml)	138.91 (49.91)	137.80 (54.54)	139.79 (47.11)
Eicosapentaenoic acid (EPA) (nmol/ml)	55.09 (48.35)	72.23 (65.69)	43.47 (27.42)*
Docosahexaenoic acid (nmol/ml)	336.36 (113.62)	342.98 (116.41)	331.53 (113.60)
*n-6*			
Sum of omega-6 fatty acids (nmol/ml)	1,199.47 (338.32)	1,098.53 (236.94)	1,270.13 (382.20)^+^
Conjugated linoleic acid (nmol/ml)	35.88 (19.34)	35.86 (11.85)	35.89 (23.28)
γ-linolenic acid (nmol/ml)	31.60 (14.56)	28.42 (13.59)	33.76 (15.01)
Dihomo-γ-linolenic (nmol/ml)	269.60 (82.57)	254.58 (66.78)	279.78 (91.39)
Arachidonic acid (AA) (nmol/ml)	861.58 (264.53)	779.66 (189.44)	918.93 (296.03)^+^
*n-6:n-3*			
Omega-6:omega-3	1.95 (0.93)	1.64 (0.56)	2.17 (1.06)*
AA:EPA	22.09 (11.1)	17.15 (9.41)	25.54 (11.09)**
**Dietary Intake**			
% taking omega-3 fatty acid supplement	41%	34%	53%
Total energy (Kcal)	2,532.03 (574.83)	2,453.16 (457.96)	2,566.74 (624.81)
Total fat (g)	103.13 (32.06)	99.47 (23.92)	104.73 (99.48)
Percent of energy from Fat (%)	36% (6%)	37% (6%)	36% (7%)
Polyunsaturated fatty acids (PUFA) (g)	22.37 (9.05)	21.31 (7.26)	22.84 (9.83)
Omega-3 fatty acids (g)	2.55 (1.15)	2.56 (0.92)	2.55 (1.26)
Saturated fatty acids (SFA) (g)	37.06 (15.76)	35.82 (7.61)	27.60 (18.36)
Monounsaturated fatty acids (MUFA) (g)	35.69 (11.20)	34.49 (9.39)	35.89 (12.19)
PUFA: SFA	0.68 (0.28)	0.63 (0.15)	0.70 (0.33)
**Inflammation (raw values)**			
IL-6 (pg/ml)	1.65 (0.76)	1.45 (0.67)	1.78 (0.80)
TNF-α (pg/ml)	11.38 (3.48)	9.34 (3.09)	12.39 (3.07)**

*AA, Arachidonic Acid; EPA, Eicosapentaenoic Acid; IL-6, Interleukin-6; TNF-α, Tumor Necrosis Factor-alpha; PUFA, Polyunsaturated Fatty Acids; SFA, Saturated Fatty Acids; MUFA, Monounsaturated Fatty Acids.*

*Cytokine concentrations presented in this table are raw values, to aid in interpretation; these were log-transformed prior to analysis. In preliminary analyses, the ADHD and Control groups were compared to one another using t-tests or chi squared tests. Significant and marginally significant groups differences based on these analyses are denoted in the table:*

+ *p < 0.10,*

* *p < 0.05,*

** *p < 0.01*.

Our hypotheses were tested using a series of linear regressions. To examine whether there were ADHD group differences in the n-6:n3 (Hypothesis 1), we ran two regression models. In the first model, the plasma n-6:n-3 was regressed on ADHD status and on a variable that captured whether the participant was taking a fatty acid supplement or not. In the second model, the plasma AA:EPA was regressed on ADHD status and fatty acid supplement status. Two regressions were used to test Hypothesis 2 (whether there are ADHD-associated differences in cytokine levels), one where TNF-α was regressed on ADHD status and on fatty acid supplementation status and pre-pregnancy BMI, and a second where IL-6 was regressed on the same variables. In four additional models, we examined the association between the n-6:n-3 and cytokine levels, where (a) TNF-α was regressed on the n-6:n-3, (b) TNF-α was regressed on the AA:EPA, (c) IL-6 was regressed on the n-6:n-3, and (d) IL-6 was regressed on the AA:EPA. Given the dearth of research relating these metrics in pregnant individuals (particularly in the context of ADHD), these models were run once without covariates and a second time, controlling for fatty acid supplementation and pre-pregnancy BMI to provide the full context for these results. To address Hypothesis 3 (whether there are ADHD-associated dietary differences), each 24-h dietary intake variable was regressed on the ADHD status variable and on fatty acid supplementation status. Additionally, concentrations of the essential fatty acids conjugated linoleic acid and α-linolenic acid were regressed on ADHD status (each essential fatty acid was considered in its own model), again controlling for fatty acid supplementation status.

## Results

### Descriptive statistics

Participants were on average 30.49 years old (SD = 5.0; range = 18–41.25 years) at enrollment. Ninety-one percent of the sample was non-Hispanic White (7% Native Hawaiian/Pacific Islander, 2% Multiple Races). The median completed education was a Bachelor's degree, with a range from some college but no degree to receiving a doctorate. See Table 1 for demographic information presented separately for the heightened ADHD symptoms and control groups.

Table 2 presents the raw means for all focal study variables, presented for the complete sample as well as separately for the heightened ADHD symptoms and control groups. Results from preliminary analyses that used *t*-tests and chi squared tests to compare the ADHD and control groups on these variables indicated that the ADHD group differed from controls for the following plasma concentrations: the heightened ADHD symptoms group had higher n-6:n-3s (*p* = 0.04), higher AA:EPAs (*p* = 0.007), lower EPA concentrations (*p* = 0.03) and higher TNF-α concentrations (*p* < 0.001). ADHD-associated increases in plasma AA (*p* = 0.06) and plasma total n-6s (*p* = 0.07) were marginally significant. None of the 24-h recall dietary data differed between heightened ADHD symptoms and control groups (*p*s > 0.33). The heightened ADHD symptoms and control groups also did not differ in the percentage of individuals who reported taking a fish oil or fatty acid supplement in the previous 30 days (34 vs. 53%, *p* = 0.18).

### Hypothesis 1: Plasma fatty acid concentrations

The results of the regressions used to test Hypothesis 1 are presented in Table 3. The heightened ADHD symptoms group had a significantly higher n-6:n-3 than controls, as indexed by both the n-6:n-3 (β = 0.30, *p* = 0.008) and the AA:EPA (β = 0.30, *p* = 0.001). These estimates are adjusted for whether the participant was taking a fish oil or fatty acid supplement, which was not significantly related to the n-6:n-3 (*p* = 0.72) but was negatively associated with the AA:EPA (β = −0.55, *p* < 0.001) in these models.

**Table 3:** Results from regression models relating ADHD status and n-6:n3 metrics and pro-inflammatory cytokines in pregnant individuals (*N* = 68).

	**Omega-6:Omega-3**		**AA:EPA**		**TNF-**α		**IL-6**	
	**β (SE)**	***p***	**β (SE)**	***p***	**β (SE)**	***p***	**β (SE)**	***p***
ADHD status^a^	0.30(0.11)	0.008	0.30(0.09)	0.001	0.35 (0.13)	0.01	0.03(0.15)	0.83
Fatty acid supplement status^b^	0.06(0.18)	0.72	−0.55(0.08)	<0.001	−0.03	0.79	0.02(0.15)	0.90
Pre-pregnancy BMI	–	–	–	–	0.35 (0.11)	0.001	0.44(0.13)	<0.001

a *0 = Control, 1 = Heightened ADHD Symptoms.*

b *0 = not taking a fish oil or fatty acid supplement, 1 = taking a fish oil or fatty acid supplement.*

*BMI, body mass index; AA, Arachidonic Acid; EPA, Eicosapentaenoic Acid; TNF-α, Tumor Necrosis Factor-alpha; IL-6, Interleukin-6.*

*Results of these regression models suggest that individuals in the heightened ADHD symptoms group had, on average, higher ratios of omega-6-to-omega-3 fatty acids (as measured by the total ratio of total omega-6s to total omega-3s, as well as by the ratio of AA to EPA), and that they had higher mean concentrations of TNF-α*.

In supplemental analyses, we also examined whether the ADHD-associated differences in the n-3 and n-6 fatty acids listed in Table 2 were still present when we controlled for fatty acid supplementation. Results indicate that plasma EPA concentrations were lower (β = −0.24, *p* = 0.017) and that plasma AA concentrations were higher (β = 0.29, *p* = 0.01) among individuals with ADHD, relative to controls. There were no significant differences in concentrations of total n-3s or total n-6s, or in any of the other individual fatty acid (*p*s ranged from 0.16 to 0.98) when supplementation was considered.

### Hypothesis 2: Plasma cytokine concentrations

**Figure 1:**
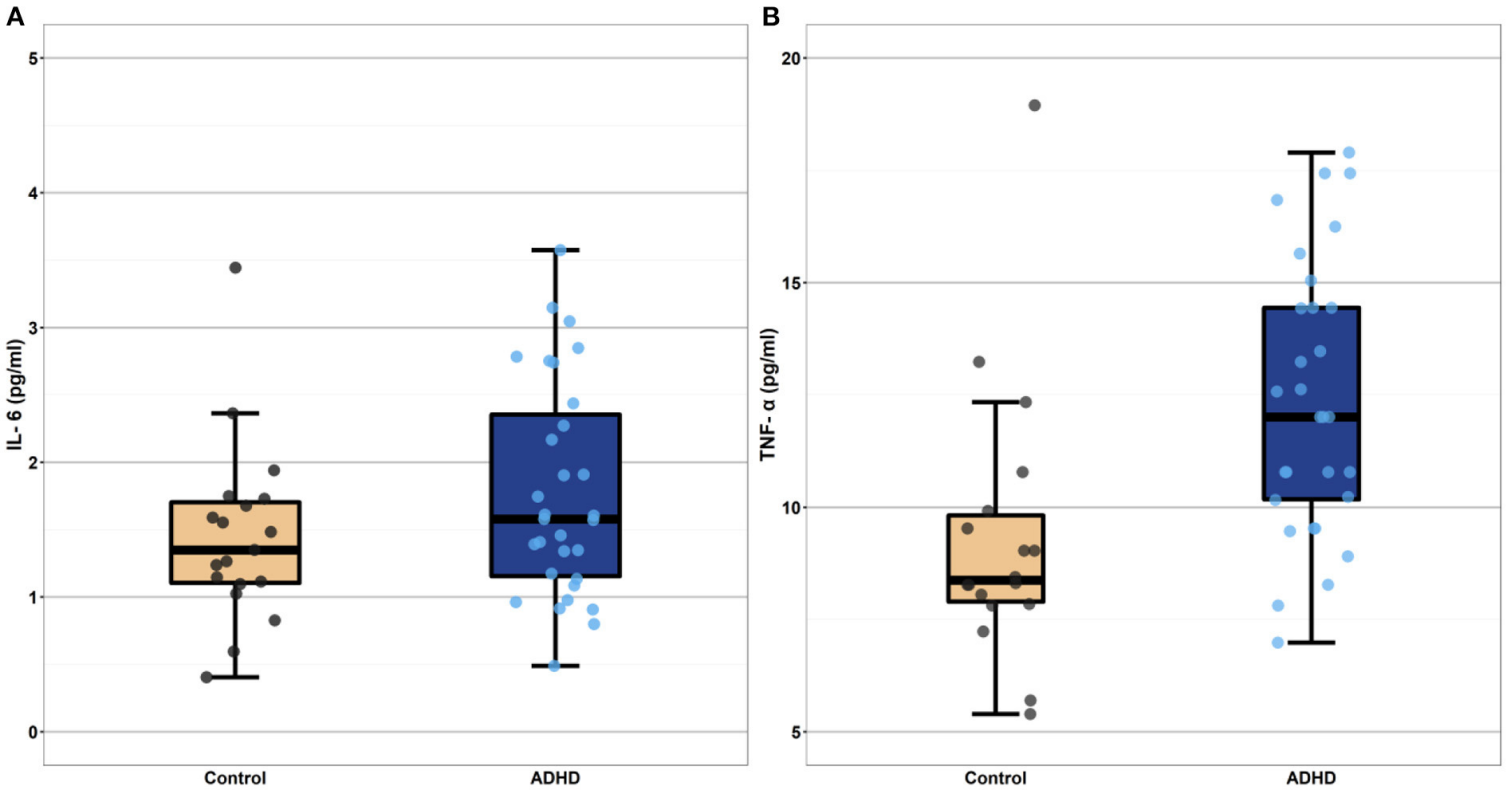
Boxplots depicting mean level cytokine concentrations in the heightened ADHD symptoms and control groups (Panel A depicts IL-6 values and Panel B depicts TNF-a values). Cytokine concentrations depicted in these boxplots are raw values, selected for ease of interpretation. These raw values were log-transformed prior to all analyses. The heightened ADHD symptoms and control groups differed significantly in their mean TNF-α concentrations (*p* < 0.001) even after controlling for participant pre-pregnancy BMI. Mean IL-6 concentration did not differ between the ADHD and control groups (*p* = 0.83). IL-6, interleukin-6; TNF-α, tumor necrosis factor-alpha.

As can be seen in Table 3 and in Figure 1, TNF-α concentrations (β = 0.35, *p* < 0.001) were higher in individuals with heightened ADHD symptoms, even after controlling for both fatty acid supplementation status (β = −0.03, *p* = 0.79) and pre-pregnancy BMI (β = 0.35, *p* = 0.001). Contrary to expectation, IL-6 concentrations were not significantly different between the heightened ADHD symptoms and control groups (β = 0.03, *p* = 0.83) in this model, though pre-pregnancy BMI was associated with higher IL-6 concentrations (β = 0.44, *p* < 0.001), as expected.

**Figure 2:**
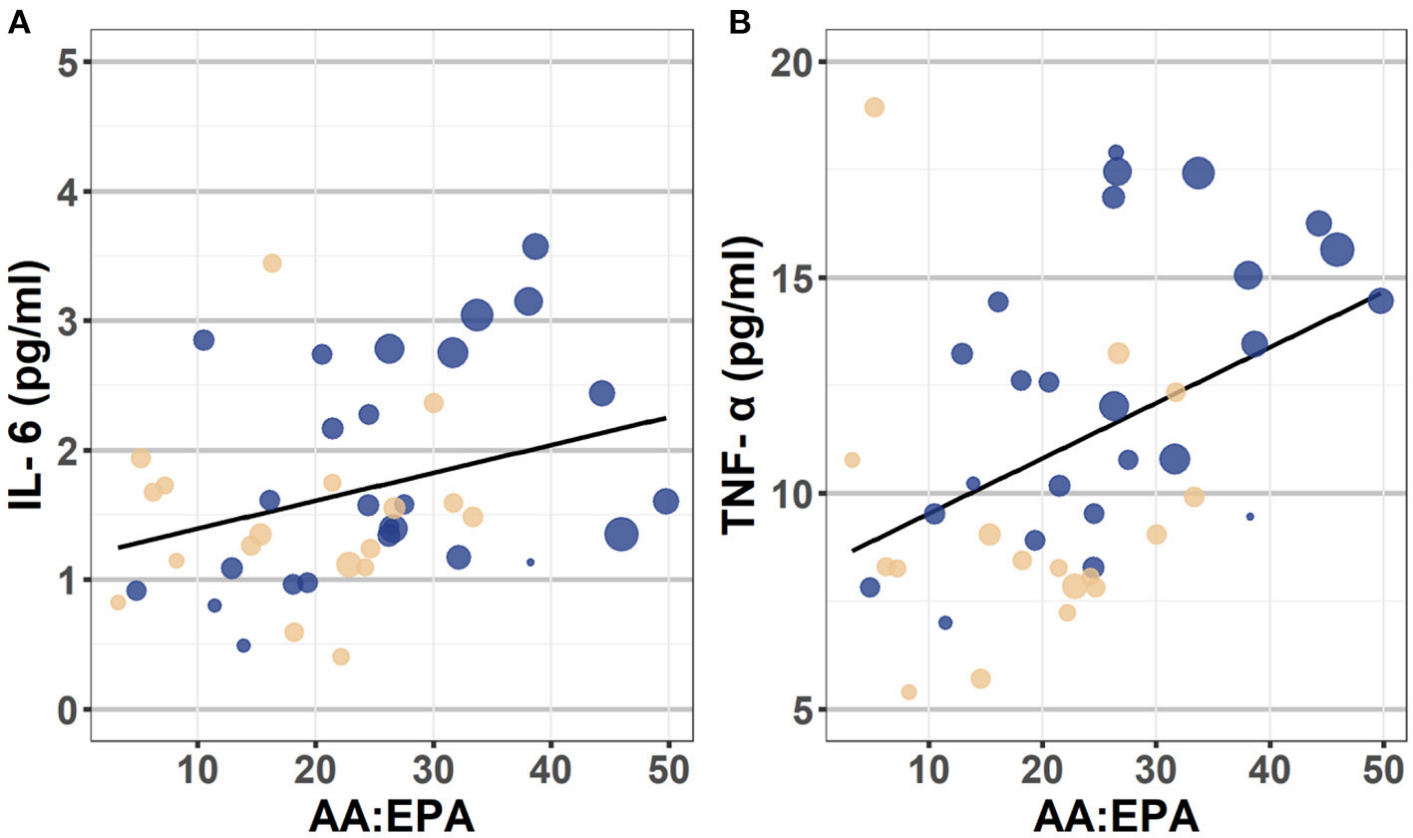
Scatterplot depicting the association between AA:EPA ratio and IL-6 (Panel A) and TNF-α (Panel B). These scatterplots depict the bivariate association between the AA:EPA and plasma concentrations of IL-6 (on the left) and TNF-α (on the right). The individual data points are color coded to reflect participant ADHD status (Yellow = Control, Blue = heightened ADHD symptoms). The diameter of each circle reflects participant pre-pregnancy BMI values, with larger circles indicating individuals with higher BMI than smaller diameter circles. Though BMI was treated as a continuous variable in analyses, they were categorized into following categories for the purposes of this plot: Healthy = 18.5–24.9, Overweight = 25.0–29.9, and Obese = 30.0 and above categories. AA, arachidonic acid; EPA, eicosapentaenoic acid; IL-6, interleukin-6; TNF-α, tumor necrosis factor-alpha; BMI, body mass index.

As presented in Table 4 and depicted in Figure 2, in a model that does not include covariates, the AA:EPA ratio was associated with greater IL-6 (β = 0.32, *p* = 0.004) and with greater TNF-α concentrations (β = 0.43, *p* = 0.001). However, these associations did not survive after controlling for BMI and fatty acid supplement use (though, as expected, BMI was significantly associated with cytokine concentrations in these models, *p*s <0.002). The n-6:n-3 was not significantly associated with IL-6 or TNF-α concentrations in either model.

**Table 4:** Results from regression models relating omega-6:omega3 metrics and pro-inflammatory cytokines (*N* = 68).

	**TNF-**α		**IL-6**	
	**β (SE)**	***p***	**β (SE)**	***p***
**Models Considering** **ΣOmega-6:ΣOmega-3**				
**Model 1: Without Covariates**				
Omega-6:omega-3	0.03 (0.02)	0.21	0.11 (0.12)	0.39
**Model 2: With Covariates**				
Omega-6:omega-3	0.10 (0.12)	0.39	0.01 (0.10)	0.94
Fatty acid supplement status^a^	−0.09 (0.14)	0.51	0.02 (0.14)	0.91
Pre-pregnancy BMI	0.44 (0.11)	<0.001	0.46 (0.13)	<0.001
**Models Considering AA:EPA**				
**Model 3: Without Covariates**				
AA:EPA	0.43 (0.12)	0.001	0.32 (0.11)	0.004
**Model 4: With Covariates**				
AA:EPA	0.22 (0.16)	0.16	0.08 (0.14)	0.58
Fatty acid supplement status^a^	0.01 (0.15)	0.94	−0.01 (0.17)	0.99
Pre-pregnancy BMI	0.37 (0.12)	0.002	0.40 (0.12)	0.001

*AA, Arachidonic Acid; EPA, Eicosapentaenoic Acid; TNF-α, Tumor Necrosis Factor-alpha; IL-6, Interleukin-6.*

a *0 = not taking a fish oil or fatty acid supplement, 1 = taking a fish oil or fatty acid supplement. BMI, body mass index. Results of these regression models suggest that while the ratio of AA-to-EPA was associated with greater TNF-α and IL-6 in models that did not considered covariates, these associations were no longer significant after controlling for pre-pregnancy BMI and whether the individual was taking a fatty acid supplement*.

### Hypothesis 3: Dietary intake measures

The heightened ADHD symptoms and control groups did not differ from one another on any of the 24-h recall dietary intake measures (total calories, total fat, percent of calories from fat (%), polyunsaturated fatty acids, n-3 fatty acids, saturated fatty acids, and the ratio of polyunsaturated fatty acids to saturated fatty acids), *p*s ranged from 0.20 to 0.94. They also did not differ in essential fatty acid concentrations in plasma (conjugated linoleic acid *p* = 0.90, α-linolenic acid *p* = 0.81).

## Discussion

Utilizing data from a unique sample of pregnant individuals, the current study examined whether there are ADHD-associated differences in polyunsaturated fatty acid or pro-inflammatory cytokine concentrations during pregnancy, a developmental period when maternal fatty acid status and inflammation have implications for the health of both the pregnant person and the developing child (27, 56–59). Results suggest that pregnant individuals with heightened ADHD symptoms, on average, had higher plasma n-6:n-3s and higher plasma concentrations of TNF-α, when compared to controls. The two groups did not differ in their dietary intake of fatty acids or other relevant nutrients, nor did they differ in concentrations of essential fatty acids (those that can only come from diet). While not conclusive, these results are consistent with the hypothesis that the observed differences in the plasma n-6:n-3 are the result of ADHD-associated differences in fatty acid metabolism, rather than simply reflecting differences in dietary intake.

The current study found that pregnant individuals with ADHD differed from controls in the n-6:n-3 using two metrics that have been used in previous research, the sum of total n-6s divided by the sum of total n-3s and the ratio of arachidonic acid to eicosapentaenoic acid concentrations (AA:EPA). Unsurprisingly, results were similar across these metrics. The observed ADHD group differences in the n-6:n-3 persisted even after controlling for whether the participant was taking a fish oil or fatty acid supplement or not. Despite n-3 supplementation being well-studied as a potential complementary treatment for ADHD (1, 60), few previous studies examining ADHD associated differences in n-3s or the n-6:n-3 have controlled for supplementation in their analyses.

The ADHD and control groups did not differ reliably in their self-reported 24-h dietary recalls of PUFA intake (total PUFAs and total n-3 fatty acids) or on other relevant nutrients, including total calories, total fat, MUFAs, SFAs and the PUFA:SFA ratio. There also were no reliable differences in plasma concentrations of conjugated linoleic acid and α-linolenic acid, fatty acids that can only come from dietary intake. Though not conclusive, our observation that there are differences in the plasma n-6:n-3 in the absence of differences in dietary intake is consistent with the purported ADHD-associated differences in the metabolism of fatty acids or in the conversion of essential fatty acids to longer-chain fatty acids (29). For example, single nucleotide polymorphisms in the fatty acid desaturase enzyme which have been shown to modulate circulating n-3 levels (61) have been associated with ADHD (30).

This study also examined whether pro-inflammatory cytokine concentrations differed between pregnant individuals with and without ADHD. We found that pregnant individuals with heightened ADHD symptoms, on average, had higher plasma concentrations of TNF-α, relative to controls. Previous research has shown that heightened gestational inflammation is associated with other types of psychiatric symptoms during pregnancy (e.g., anxiety, depression) (42, 51), but this is the first study to report that pregnant individuals with heightened ADHD symptoms have greater concentrations of pro-inflammatory cytokines. We did not see differences in plasma IL-6 between pregnant individuals with and without heightened ADHD symptoms, though this study may not have been adequately powered to detect such an effect. This is in contrast to previous research which reports differences in IL-6 between children with and without an ADHD diagnosis (38–40), but is consistent with findings from Corominas-Roso et al. which reports that adults with and without ADHD did not differ from one another in serum IL-6 concentrations (though, of note, these authors also did not find a differences in serum TNF-α) (62).

Consistent with the assertion that a greater n-6:n-3 is indicative of a more pro-inflammatory state, we found a positive association between the AA:EPA ratio and plasma concentrations of both IL-6 and TNF-α in models that did not include covariates. However, these effects did not survive controlling for pre-pregnancy BMI. Interestingly, in their study of pregnant individuals with prenatal depression, Chang et al. (12) similarly did not find a correlation between the n-6:n-3 and TNF-α, despite finding that the n-6:n-3 and TNF-α were increased in depression. The fact that individuals with ADHD are at heightened risk for obesity [see (63) for a systematic review]—which could be the long-term consequence of an increased n-6:n-3 rather than simply a confound–makes it challenging to fully interpret these findings.

The precise mechanistic connection between ADHD, PUFAs and inflammation is not fully elucidated, but see (64) for a comprehensive review on the association between PUFAs and inflammation. Fatty acids are critical nutrients in maintaining neuronal signaling (65). Additionally, the n-3 PUFA membrane content has been shown to influence neurotransmitter receptor functionality in dopaminergic neurons (66) which has implications for behaviors related to ADHD. In glial cells, levels of n-3 PUFAs can influence microglial phagocytosis (67) as well as astrocyte differentiation (68). Notably, microglia are the main cell type able to detect and respond to inflammatory signals in the brain. Further, microglia express several inflammatory receptors that have also been suggested to be sensitive to lipids (69). Animal models show that n-3 PUFA supplementation can inhibit microglial activation and the expression of pro-inflammatory cytokines, including TNF-a and IL-6 (70). It is plausible that altering microglial functional states *via* the n-6:n-3 in the brain may provide a connection between ADHD, PUFAs, and inflammation.

This study had a number of strengths. This is the first study to examine fatty acid or cytokine differences in pregnant individuals with and without heightened ADHD symptoms. Though differences in n-6:n-3 and pro-inflammatory cytokine concentrations have been observed in children with ADHD (6, 38, 39), no previous study has examined these associations during pregnancy, despite the profound implications that fatty acids and cytokines during pregnancy have for both the health of the pregnant person and for fetal development (26–28, 71). Methodological strengths of this study include assessment of fatty acids in plasma, the use of 24-h recall data collected by trained dietitians, and our inclusion of covariates that may impact fatty acid status and inflammation (most studies in this area have not considered covariates in their analyses).

This study also had limitations, including our small sample size. Though samples of this size are common in this literature, we may have lacked sufficient power to detect some effects, for example a difference in plasma IL-6 based on ADHD status. Thus, the null findings in this study should be viewed as inconclusive. In this first study investigating these associations in pregnant individuals, we focused on plasma concentrations of fatty acids, which reflect current fatty acid levels. Future studies on this topic should also examine fatty acid levels in red blood cell membranes, which reflect more chronic (i.e., the previous 3 months) levels of fatty acids. Examination of other plasma lipid levels (i.e., triglycerides) during pregnancy and may also yield important insights. While the unique nature of our sample (of pregnant individuals living in the US) is a strength of this study, it may limit generalizability of our findings. Future research should examine these associations utilizing data from participants residing in other countries as well as in non-pregnant populations. We examined two pro-inflammatory cytokines in our analyses (selected because they were shown to be related to ADHD in previous research) that were assessed at one timepoint in pregnancy. Future research should examine a wider range of relevant immune factors, assessed at multiple times in pregnancy. While our study included measurement of ADHD symptomatology assessed using a well-validated and widely used scale with established norms, we did not assess ADHD diagnosis, which also may limit the generalizability of our findings. Last, while metrics such as the n-6:n-3 offer a more comprehensive picture of fatty acid status than absolute values of n-3s or n-6s, the n-6:n-3 is also not a perfect or complete measure of fatty acid status. For example, trans fatty acids interfere with the desaturation and elongation of both omega-6 and omega-3 fatty acids (10). Future research should include other such metrics (including measures of SFAs, MUFAs, and measures of PUFAs not examined here, including docosapentaenoic acid) in their analyses.

## Summary and conclusion

This study found that, on average, pregnant individuals with heightened ADHD symptoms had higher plasma n-6:n-3s and higher TNF-α concentrations in plasma relative to controls. The heightened ADHD symptoms and control groups did not differ statistically on self-reported dietary intake (assessed using 24-h recalls), nor on essential fatty acid concentrations in blood. Though not conclusive, these findings are consistent with a picture of altered fatty acid metabolism in ADHD.

## Data availability statement

The raw data supporting the conclusions of this article will be made available by the authors, without undue reservation.

## Ethics statement

All study procedures were reviewed and approved by the Oregon Health & Science University Institutional Review Board. Written informed consent to participate in this study was provided by the participants.

## Author contributions

ES and JN conceived the project. HG, ES, JN, and JL designed the research. HG, ES, and JL analyzed the data. AM data created the plots. HG and ES wrote the manuscript, with contributions from JN, JL, GD, AM, and KH. All authors discussed the data. All authors contributed to the article and approved the submitted version.

## Funding

Research reported in this publication was supported by the Abracadabra Foundation, the Bob and Charlee Moore Institute for Nutrition and Wellness, and by the National Institutes of Health under National Institute of Mental Health award numbers R3759105 (JN), R01MH117177 and R01MH124824 (ES and JN), K01MH120507 (HG), and R01MH117177-S1 (ES and JN). This material is the result of work supported with resources and the use of facilities at the VA Portland Health Care System and OHSU, Portland, OR.

## Conflict of interest

The authors declare that the research was conducted in the absence of any commercial or financial relationships that could be construed as a potential conflict of interest.

## Publisher's note

All claims expressed in this article are solely those of the authors and do not necessarily represent those of their affiliated organizations, or those of the publisher, the editors and the reviewers. Any product that may be evaluated in this article, or claim that may be made by its manufacturer, is not guaranteed or endorsed by the publisher.

## Author disclaimer

The content is solely the responsibility of the authors and does not necessarily represent the official views of the National Institutes of Health. Contents do not represent the views of the U.S. Department of Veterans Affairs or the United States Government.
